# Loneliness by Design: The Structural Logic of Isolation in Engagement-Driven Systems

**DOI:** 10.3390/ijerph22091394

**Published:** 2025-09-06

**Authors:** Lauren Dwyer

**Affiliations:** School of Communication Studies, Faculty of Business, Communication Studies, and Aviation, Mount Royal University, Calgary, AB T3E 6K6, Canada; ldwyer@mtroyal.ca

**Keywords:** loneliness, digital technologies, artificial intelligence, design, public health, digital public health

## Abstract

As the prevalence of public discourse pertaining to loneliness increases, digital interventions, such as artificial intelligence companions, are being introduced as methods for fostering connection and mitigating individual negative experiences of loneliness. These tools, while increasing in volume and popularity, operate within and are shaped by the same engagement-driven systems that have been found to contribute to loneliness. This meta-narrative review examines how algorithmic infrastructures, which are optimized for retention, emotional predictability, and behavioural nudging, not only mediate responses to loneliness but participate in its ongoing production. Flattening complex social dynamics into curated, low-friction interactions, these systems gradually displace relational agency and erode users’ capacity for autonomous social decision making. Drawing on frameworks from communication studies and behavioural information design, this review finds that loneliness is understood both as an emotional or interpersonal state and as a logical consequence of hegemonic digital and technological design paradigms. Without addressing the structural logics of platform capitalism and algorithmic control, digital public health interventions risk treating loneliness as an individual deficit rather than a systemic outcome. Finally, a model is proposed for evaluating and designing digital public health interventions that resist behavioural enclosure and support autonomy, relational depth, systemic accountability, and structural transparency.

## 1. Introduction

Reports of loneliness have surged since the onset of the COVID-19 pandemic, drawing renewed attention to what public health officials now describe as a global crisis [1]. Recent policy analyses underscore that loneliness is increasingly framed not only as a personal health issue but also as a policy concern, with clear technological dimensions [2,3]. Goldman and colleagues [2] provide a cross-national review of loneliness and social isolation policies, noting that many governments now explicitly include technology-based interventions in their strategic plans. In the UK, additional studies by Jentoft and colleagues show how political discourse around loneliness often situates older adults within narratives of digital inclusion, even as these narratives risk oversimplifying structural inequities [3,4]. In 2020, 54% of Canadians and 36% of Americans reported heightened feelings of isolation, a marked increase from previous years [5,6]. Hawkley [7] similarly emphasizes that public policy must grapple with both the social determinants of loneliness and the role of emerging technologies in shaping them. Although loneliness is not formally classified as a mental illness in the Diagnostic and Statistical Manual of Mental Disorders (DSM-5-TR) [8], it is deeply entangled with mental health outcomes, including depression [9], and is linked to significant physical health risks such as cardiovascular disease and early mortality [10]. Developmental perspectives add further nuance, as Hang et al. [11] highlight how chronic loneliness can take root early in life, with digital environments playing a formative role in either buffering or exacerbating social disconnection. Now described as a “parallel pandemic,” the crisis of disconnection has persisted even as the stigma surrounding loneliness has shifted in its cultural weight [12,13]. In response, digital intervention (i.e., artificial intelligence companions, chatbots, and social wellness apps) have emerged as scalable, accessible tools that have shown promise in fostering emotional support and facilitating connection, particularly for older adults and geographically or socially isolated individuals [13,14,15,16,17,18,19].

Despite these advantages, the literature on human–computer interaction (HCI) and design studies suggest the same digital infrastructures designed to alleviate loneliness are implicated in its ongoing production [20,21,22,23,24,25,26]. This paradox highlights a deeper tension at the intersection of public health, design, and algorithmic media: while AI systems may mediate individual experiences of disconnection, they are also structurally embedded within platform logics that prioritize engagement, predictability, and behavioural nudging over relational depth. In other words, the systems offering therapeutic solutions are often architected around the same imperatives that displace meaningful social interaction in the first place.

The roots of this contradiction lie, in part, in transformations to how information is filtered, circulated, and experienced in digital life. Gatekeeping theory, once central to media and communication studies, described the editorial decisions made by individuals, journalists, broadcasters, editors about what entered the public sphere [27]. The gate was visible and accountable, albeit imperfectly. Today, as algorithmic systems increasingly determine what we see, believe, and engage with, gatekeeping has become computational. It is no longer a social negotiation but a proprietary operation encoded into the infrastructure of digital platforms. As Tarleton Gillespie notes [28,29], choices made by designers working within commercial ecosystems embed algorithmic systems with values like frictionless navigation to minimize frustration and challenges in the digital sphere. These values are not neutral; they shape what rises to visibility and what disappears into algorithmic oblivion. In this sense, platforms do not merely mediate attention, as they actively structure epistemological and emotional life.

The result is a digital media environment where visibility becomes synonymous with legitimacy [30], and emotional salience rather than informational value determines what circulates. Zuboff’s concept of surveillance capitalism [31] and Casas-Cortés and colleagues’ concept of platform capitalism [32] explain how these systems move beyond the passive prediction of behaviour toward its active shaping. In this environment, reality itself becomes what is most clickable, most shareable, and most emotionally intense. Tufekci [33] illustrates how attention is redirected by rewarding engagement over accuracy, pulling users toward more polarizing or addictive experiences. In this context, scholars have argued that loneliness may be more than a byproduct of digital life, suggesting that it is shaped and, in some cases, reinforced by dominant design logics embedded in platform capitalism [34,35,36]. The same algorithmic infrastructures that provide social recommendation, affirmation, or simulated empathy are often optimized to fragment user attention [33], narrow emotional range [31], and displace complexity in favour of simplified, coherent narratives [29,37]. Where earlier generations encountered propaganda as deliberate and centralized, today it is ambient, embedded in interface design, emotional profiling, and the algorithmic ordering of experience. Targeting is no longer demographic but psychographic, functioning through predictive emotional calibration.

This review examines the research surrounding loneliness not only as an emotional or interpersonal state but as a structural consequence of the technologies designed to treat it. Drawing on the fields of communication and media studies, HCI, design, and public health, the analysis examines how algorithmic infrastructures mediate, perpetuate, and produce loneliness within the logics of platform capitalism. It explores how personalization, artificial intimacy, and engagement-maximizing design displace agency and relationality, while simultaneously offering interventions that treat loneliness as an individual pathology. Finally, we propose a human-centered, ethically grounded framework for designing AI and digital public health tools that resist behavioural enclosure, support relational autonomy, and center human flourishing in an age of algorithmic control.

## 2. Methodology

This study follows a meta-narrative review methodology, guided by the Realist and Meta-narrative Evidence Synthesis (RAMESES) framework [38,39]. The meta-narrative approach was selected for its suitability in examining how different research traditions (public health, communication studies, behavioural design, and human–computer interaction (HCI)) and their respective epistemic cultures conceptualize loneliness in the context of digital and technological design [40]. This method prioritizes pluralism, reflexivity and historicity over a single standard of evidence, enabling insights to be considered from diverse epistemic traditions that rarely intersect in conventional systematic reviews [40].

### 2.1. Research Questions

This review is guided by two central questions:

1. How is loneliness framed in the fields of digital and technological design (including HCI and communication studies), compared to its clinical and public health representations?
2. How can these perspectives inform the design of ethical digital public health interventions?

### 2.2. Rationale for Review Type

The meta-narrative review methodology was chosen for its comparative mapping of concepts, theories, and evidence across distinct epistemic traditions. The RAMESES framework provides guidance for integrating differing perspectives to identify conceptual convergences and divergences, ultimately informing a new evaluative framework for digital public health designs [38,39].

### 2.3. Search Strategy

An exploratory scoping phase was undertaken during April and May of 2025 to familiarize the researcher with the breadth of the literature and terminology used across the relevant fields. This initial review consisted of informal browsing of electronic databases, citation chasing of seminal works, and informal consultations with experts in public health, communication studies, and human–computer interaction. This stage identified and helped to draw connections and distinctions between the key epistemic traditions being explored in this review: public health/clinical research, behavioural science, communication and media studies, and design/technology studies. This work informed the iterative development of search terms and inclusion/exclusion criteria.

The formal search strategy combined electronic database searches, hand-searching of reference lists from selected articles for additional relevant publications, and a grey literature review of policy reports, white papers, and industry documentation from organizations such as the WHO, OECD, and various national public health agencies. The following databases were searched using university access: PubMed, APA PsycInfo, Communication & Mass Media, ACM Digital Library. Additional searches were run through Google Scholar. These databases were chosen to ensure coverage across the health, psychology, social science, and technology literature.

Search terms were combined using Boolean operators and adapted for each database.

Initial terms and concepts included the following: *Loneliness* OR *social isolation* AND *Digital public health* OR *digital intervention* OR *AI companion* OR *social robot* OR *mental health app* OR *wellness app*; *Design paradigm* OR *algorithmic infrastructure* OR *platform capitalism* OR *engagement-driven* OR *behavioural nudging* OR *artificial intimacy.*

Boolean operators and truncations were adapted for each database. Searches were limited to works published between January 2010 and May 2025, in English, to capture the literature reflecting the rise of algorithmically mediated social environments and the proliferation of digital health interventions. Where available on database searches, the peer reviewed and references available filters were applied.

Initial results yielded a combined 7786 articles, books, and grey literature. Searches were re-run at key points in the review to incorporate newly published studies and with considerations given to the evolving nature of new technologies. Citation trails were followed from influential works to better understand the narratives of each field.

### 2.4. Inclusion/Exclusion, Screening and Selection Processes

Inclusion criteria: Articles consisted of empirical studies, theoretical papers, systematic reviews, policy analyses, or critical essays engaging with loneliness in the context of digital or technological mediation. Works examining the design logic, algorithmic mediation, or infrastructural dynamics of digital platforms relevant to loneliness were included. Studies contributing conception or empirical insights into public health, communication, or design implications were also included.

Exclusion criteria: Studies on loneliness with no reference to digital or technological systems were not included; however, seminal texts that define loneliness were initially referenced to define the field. Interventions limited to traditional telehealth without algorithmic or engagement-driven features were not included. Opinion pieces lacking substantive engagement with the existing literature were not included.

Selection was guided by pragmatism, with sources being retained only if they were likely to inform cross-disciplinary sense making. Quality appraisal was tradition-specific: public heath articles were assessed for methodological rigour using health sciences standards; design and communication studies were judged according to qualitative and theoretical scholarship criteria; and human–computer interaction research was considered under quantitative methodological standards.

**Figure 1:**
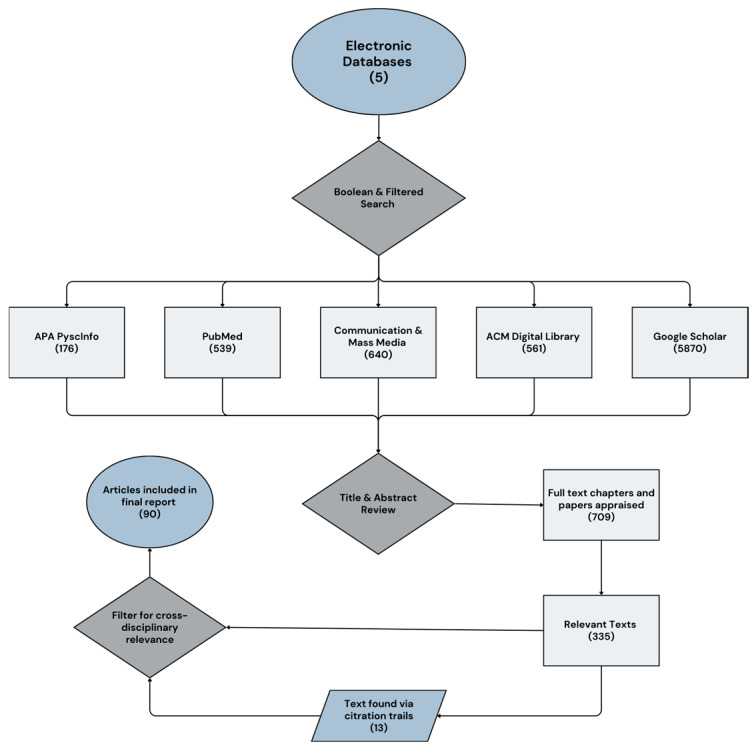
RAMESES search criteria and screening process.

All retrieved records were imported into reference management software (Zotero 7.0.24), where duplicates were removed. Titles and abstracts were screened for relevance, and potentially relevant records proceeded to full-text review, during which inclusion and exclusion decisions were documented alongside justifications. Iterative searching occurred throughout to refine search terms to those discovered in included works and cross-citation mapping between traditions. Figure 1 summarizes the search, screening, and selection process, including database searching, grey literature, and citation chasing.

Data extraction was designed to support meta-narrative cross-disciplinary sense making rather than mechanical coding. Synthesis followed the RAMESES principles by prioritizing sources most useful for building cross-disciplinary understanding; evaluating each tradition’s sources based on their own quality criteria and epistemic assumptions, considering the traditions’ evolutions over time; informal peer review through discussions with colleagues with relevant expertise but outside of the researcher’s primary discipline and maintaining awareness of the researcher’s own positionality as a communications and design scholar with a background in artificial intelligence and social robotics.

## 3. Analysis and Results

Cross-disciplinary analysis of the literature identified four principal research traditions that engage with loneliness in the context of digital and technological design: (1) Public Health and Clinical Epidemiology, (2) Behavioural Science and Psychology, (3) Human–computer Interaction (HCI) and Design Research, and (4) Communication and Media Studies. Within these traditions, the meaning of loneliness and the factors deemed most salient to its emergence and the perceived role of digital interventions varied substantially. The following synthesis outlines the prevailing conceptualizations, with comparisons made to identify commonalities, divergences, and opportunities for cross-disciplinary integration. Table 1 below outlines these findings. A systems thinking approach was applied to bridge these gaps, treating technological design, social practices, and policy environments as interlinked components of a single dynamic system. This perspective was used to consider the feedback loops between technological affordances, user behaviours, and psychosocial outcomes as well as account for multi-level determinants of loneliness such as interface features and community infrastructure. Potential unintended consequences of engagement-driven designs offering short-term relief but deepening long-term disconnection formed the basis for this article’s proposed discussion between these traditions.

The following discussions build on this synthesis by unpacking four thematic domains that cut across traditions: (1) the hegemony of digital design paradigms, (2) the role of algorithmic infrastructures as mediators and producers of loneliness, (3) structural logics of platform capitalism and algorithmic control, (4) the convergence and tensions between public health and design perspectives. A fifth theme emerged in the readings, suggesting a framework for priorities for (5) a digital public health design framework. This progression moves from critical examination of underlying structures to the development of an integrative framework that preserves the conceptual integrity of each tradition while offering a coherent, ethically grounded approach to digital public health design. A table noting the selected articles, their tradition, and thematic domains can be found in the Supplemental Materials, Table S1.

## 4. The Hegemony of Digital Design Paradigms

### 4.1. Hegemonic Digital Infrastructures and the Structuring of Loneliness

The influence of structural logics as infrastructures on social experience reflects deeper questions about agency in the digital age [30,35]. Ågerfalk [41] describes AI as a form of digital agency, mediating and even substituting human decision making in ways that reconfigure relational autonomy, which Ho [42] defines as the ability to act meaningfully within relationships while retaining independence from coercive digital mediation. Extending this, Kuss and Meske [43] and Dattathrani and De’ [44] consider how AI shifts agency from individuals to sociotechnical systems, while Burkitt’s [45] relational sociology frames agency itself as emerging from networks of human and non-human actors. Loneliness, far from being solely an emotional or psychological phenomenon, emerges systematically in the literature as having a reciprocal relationship to prevailing hegemonic paradigms in digital and technological design [13,14,15,16,17,18,19,46]. Contemporary digital infrastructures, shaped by commercial imperatives and optimization strategies, are found to embed structural logics that significantly contribute to the persistence and exacerbation of loneliness [2,7,46].

While this review focuses on shared structural logics, it is important to distinguish between the design approaches and relational impacts of three overlapping but distinct categories of digital intervention that appear in the literature: (1) AI companions and conversational agents, such as Replika, ChatGPT, or ElliQ, designed to simulate relational presence and provide emotional support; (2) digital wellness and mental health apps, which use behavioural nudges, gamified CBT, or journaling to manage mood and stress; and (3) social media platforms, which are not explicitly therapeutic but significantly shape users’ affective and social landscapes through algorithmic curation. While each of the above differs in function and intent, they operate within a shared infrastructure of platform capitalism and engagement-driven design. Despite their functional differences, each of these systems operates within infrastructures that reward predictability, data extraction, and continuous engagement. Chen et al.’s [47] work documents how adolescents encounter algorithmic features (such as infinite scroll and autoplay) that intentionally extend usage sessions while narrowing the diversity of social interaction. Studies like Chen et al. [47] demonstrate how large-scale empirical work on engagement-prolonging designs confirms these tendencies. As a result, they may contribute to a common relational outcome: digitally saturated environments that amplify disconnection, emotional flattening, and the erosion of relational autonomy [47].

Central to these paradigms is the prioritization of superficial interactions over meaningful relational depth [13,15,17]. Social media platforms, driven by commercial imperatives for maximum engagement, favour quantifiable metrics such as follower counts, likes, and frequent yet brief interactions [15,31]. This logic results in social experiences that are abundant in volume but lacking in emotional authenticity, leaving individuals digitally connected yet emotionally alienated [48,49]. The subsequent sense of disconnection illustrates a paradox central to contemporary digital design, wherein increased connectivity fails to correspond to genuine relational satisfaction or emotional fulfillment.

Digital design paradigms that subscribe to hegemonic practices are also frequently found to result in the displacement of meaningful face-to-face interactions [13,48,49]. Technologies engineered for continual user engagement inherently encourage increased screen time, inadvertently reducing opportunities for more enriching, embodied interpersonal encounters [50,51]. This displacement not only impoverishes the social experience but also diminishes users’ opportunities to engage in complex, nuanced interactions, interactions that provide emotional nourishment and relational depth not readily replicated through digital channels [48,51].

### 4.2. Simulated Intimacy and the Erosion of Relational Depth

The dominance of algorithmically driven artificial intimacy further compounds this isolation. AI companions and conversational agents, designed to provide consistently empathetic and conflict-free (i.e., frictionless) interactions, promote dependency by offering emotionally predictable relationships [15,17,50,52]. The design ethos of frictionless interactions, although comforting, can inhibit users from engaging with the complexities and emotional messiness of authentic human relationships [53,54,55]. While “frictionless” interfaces are often celebrated in commercial design, Kemper [53] critiques this as an aesthetic and philosophical orientation that erases opportunities for meaningful pause or reflection. Chen and Schmidt [56] similarly propose “positive friction” as a countermeasure, in which small, intentional points of resistance are introduced to preserve user agency and promote deeper and more critical engagement. Over-reliance on these predictable forms of artificial intimacy undermines the intrinsic relational growth that arises through genuine human friction, negotiation, and compromise, thereby intensifying users’ underlying loneliness [50,52,56].

Algorithmic designs often create an “illusion of recognition,” providing users with simulations of empathy and understanding without the reciprocal depth fundamental to authentic relationships [50]. Empirical studies of AI companions confirm the risks of these relationships; Jacobs [50] shows that reliance on AI-mediated “recognition” can shift patterns of social validation, while George et al. [52] interrogate the ethics of simulated intimacy in generative AI, warning that such systems may offer comfort at the expense of authentic reciprocity. Rather than genuinely alleviating loneliness, such monologic interactions result in what scholars’ term “digitized loneliness,” wherein individuals are effectively conversing with reflections of their own emotional states within algorithmically curated echo chambers [50,52,55,57]. Illusory intimacy, while compelling, is fundamentally incomplete and reinforces rather than resolves users’ emotional isolation [48,57].

### 4.3. Design Affordances, Sensory Deficits, and Affective Disconnection

Hegemonic digital paradigms are likewise characterized by their commercial incentives, frequently producing addictive designs intended to maximize continuous user engagement [31,32,34]. Platforms intentionally incorporate social metrics and reward mechanisms that encourage persistent social comparison through continuous presence online and an emotional dependency on digital approval [47,58,59,60]. Such designs are as commercially advantageous as they are relationally detrimental, exacerbating experiences of loneliness by discouraging users from pursuing meaningful offline connections and creating cycles of compulsive digital interaction. Concurrently, digital communication systems suffer from inherent sensory impoverishment [25,51]. Designed primarily for textual and visual communication, they lack critical relational cues such as tone of voice, body language, and nuanced emotional expressions, all of which are fundamental to deep emotional resonance [51]. This sensory deficit inevitably diminishes the emotional quality of digital interactions, rendering them less fulfilling and leaving users feeling emotionally detached despite apparent digital connectivity.

The reproduction and reinforcement of societal biases within algorithmic systems additionally contribute to loneliness [13,46,61]. AI models trained on inadequately diverse datasets risk perpetuating discriminatory views, inadvertently amplifying prejudices and contributing to the social isolation of already-marginalized groups [61]. This bias-driven amplification further entrenches social divisions, reducing opportunities for inclusive integration and meaningful cross-group social engagement [61]. 

### 4.4. Individualisation, Medicalization, and the Obfuscation of Structural Causes

Digital technology interventions, whether they be AI companions, chatbots, digital wellness and mental health apps, or social media platforms, may provide short-term relief; however, they fail to sustainably address chronic loneliness, as they inherently lack the reciprocal emotional intimacy central to long-term relational satisfaction [16]. This design misalignment fosters cycles of temporary relief followed by enduring dissatisfaction and emotional isolation. When dominant digital paradigms frequently individualize and medicalize loneliness, the hegemonic framing becomes one of individual deficit or medical condition [62]. The pervasive emphasis on individualized technological solutions for self-management, reflecting broader neoliberal ideologies, can deflect attention from underlying systemic and socioeconomic drivers of loneliness. By framing loneliness as primarily an individual responsibility, digital designs obscure necessary discussions of community structures, socioeconomic inequities, and broader collective conditions that systematically produce relational disconnection [62]. When technologies prioritize immediacy, constant connectivity, and sustained engagement, over the users’ deeper emotional needs for meaningful connection and relational authenticity [25,61], loneliness becomes a predictable outcome. The experience then reflects the logic of hegemonic digital design, driven by commercial engagement metrics, algorithmic optimization, and individualizing narratives [32,61]. Addressing loneliness effectively must require systemic reorientations in technological design, moving beyond short-term emotional validation and toward fostering relational depth, equitable access, authentic interpersonal reciprocity, and structural accountability.

## 5. Algorithmic Infrastructures as Mediator and Producer

Algorithmic infrastructures, by design, mediate contemporary social interaction by shaping both the nature and availability of interpersonal engagements [13,24,48,49,50]. These systems, encompassing AI, digital technologies, and social media platforms, play a dual role in both alleviating and intensifying loneliness, depending on the design and usage context [50,63,64].

### 5.1. Technology as Mediator

On the alleviating side, digital technologies provide meaningful pathways to social connection, which is particularly beneficial for those geographically isolated, socially marginalized, or experiencing situational loneliness [7,50,63,64]. Platforms such as online forums, social networking sites, and digital wellness apps can effectively bridge physical and metaphorical distances, creating virtual spaces where users connect over shared experiences, interests, or identities [23]. This capacity became critically evident during the COVID-19 pandemic, when telehealth platforms, virtual support groups, and social media networks offered indispensable social support amid widespread physical isolation [65]. Additionally, algorithmic personalization enhances the emotional resonance and relevance of these digital interactions [23,52]. Emotionally intelligent AI technologies, such as the social robot ElliQ, use behavioural analytics to infer emotional states, adapting their interactions to subtly encourage social engagement among older adults [23]. Likewise, AI-powered companions like Replika and even later versions of ChatGPT offer synthetic yet responsive conversational partners, designed explicitly to make users feel understood, recognized, and supported, which are crucial psychological elements identified in loneliness interventions [17,55].

### 5.2. Technology as Producer

Despite these advantages, algorithmic mediation, like all algorithmic interventions, is not inherently benign. The same technologies that enable personalized interactions also structure environments characterized by censorship in the form of computational enclosure, a narrowing of informational experience through automated affirmation and selective exposure [66,67,68]. Milli et al. [58] demonstrate how engagement algorithms amplify divisive or emotionally charged content, while Ibrahim et al. [59] identify the specific design patterns that lead to harm, including narrowing emotional range and reinforcing dependency. Building on Tufekci’s [69] account of computational agency as a narrowing of informational experience, Grabher’s Enclosure 4.0 [70] analysis of how platforms capture data and scale predictive logics, and Couldry and Mejias’s [71] framing of datafication as a form of digital enclosure, I use the term “behavioural enclosure” to describe how predictive systems quietly constrain users’ affective and behavioural horizons. Rather than explicitly forbidding certain content, platforms gradually reduce friction, ambiguity, and contradiction to preserve emotional continuity and platform retention [53,56,64]. Personalization algorithms, learning continuously from users’ behavioural data and emotional cues, systematically reinforce existing beliefs and emotional preferences, filtering out contradictory or challenging content [37,69,72]. Rather than enabling true social agency, this personalization constrains informational autonomy, creating an experience of algorithmically facilitated frictionlessness that prioritizes comfort over genuine relational growth. These dynamics raise questions of relational autonomy [42]. Unlike traditional forms of censorship that restrict content by explicit force, algorithmic enclosure subtly removes complexity, contradiction, and discomfort by rendering them less visible or less accessible [66]. Such algorithmically driven filtering risks entrenching a narrowed worldview, diminishing users’ tolerance for ambiguity, and ultimately weakening relational depth in favour of superficial engagement [61]. By prioritizing ease of interaction and emotional predictability, platforms inadvertently encourage interactions that are frequent but emotionally shallow, exacerbating rather than alleviating loneliness over time.

The dynamics of algorithmic mediation have significant implications for democratic social engagement. Personalized realities, individually curated by algorithms, undermine a collective baseline of shared information, weakening capacities for collective decision making [31,32,33]. Users no longer merely disagree; they inhabit fundamentally different informational ecosystems, constructed by distinct algorithmic logics, each sustained by their own self-reinforcing truths [27,28,29]. In this way, algorithmic infrastructures not only shape individual experiences of loneliness but influence broader social cohesion, potentially deepening feelings of alienation and disconnection.

Critical perspectives highlight that reliance on digital solutions such as AI companions can lead to dependence on the appeasement of the system, potentially eroding users’ motivation and ability to engage authentically in offline human relationships [50]. The risk here lies in digital relationships supplanting rather than supplementing genuine human connections, potentially leaving users feeling superficially connected yet fundamentally isolated [52,68].

Algorithmic mediation also raises critical issues of access and equity. Socioeconomic status, age, cultural context, and digital literacy significantly affect individuals’ ability to benefit from digital interventions [49]. The result is an ambivalent landscape in which, as Cahyono and Adiawaty [49] observe, the same technologies that promise to connect us often operate in ways that entrench isolation. Without careful attention to these structural barriers, algorithmic infrastructures may inadvertently reinforce existing inequalities, marginalizing those who stand to gain the most from meaningful digital connections.

## 6. Structural Logics of Platform Capitalism and Algorithmic Control

### 6.1. Platform Capitalism and the Infrastructure of Loneliness

The structural logics that make up the scaffold of what Casas-Cortés et al. [32] define as “platform capitalism,” are a mode of economic organization where value is extracted from social interactions themselves, and in what Nowotny [26] terms the “illusion of control” in predictive algorithms, which obscures the asymmetry between user agency and platform power. Platform capitalism and algorithmic control fundamentally shape both the lived experience of loneliness and the dominant technological responses proposed to address it. These algorithms are characterized by imperatives of scalability, engagement maximization, and data extraction that prioritize profit over well-being by embodying the specific values and priorities that often conflict with the conditions necessary for meaningful social connection [18,62]. In the context of digital loneliness interventions, such logics offer the appearance of connection and care, while frequently reproducing or exacerbating the very forms of isolation they claim to remedy.

At the center of platform capitalism lies the pursuit of profit through engagement [31]. Commercial platforms are incentivized to design technologies that prioritize user retention and behavioural predictability, often through the use of engagement-prolonging features (EPFs) such as infinite scroll, autoplay, and social nudging mechanisms [20,52,61,73]. These mechanisms range from profile view alerts to interaction streaks and leverage social anxieties and cognitive biases to maintain user presence rather than to foster relational depth. Within this model, attention is a commodity, and loneliness becomes an opportunity for monetization, giving rise to what has been described as a “billion-dollar loneliness industry” [62,74]. As Ruckenstein [24] argues, algorithmic systems are not neutral mediators but active producers of affect, shaping how users feel and act in order to sustain engagement [36]. This builds on Pariser’s [37] “filter bubble” and Couldry and Mejas’s [71] “costs of connection,” both of which describe how datafication transforms social life into a resource for extraction. Emotional vulnerability, far from being addressed, is instrumentalized as a means of generating value.

### 6.2. Affective AI and the Commodification of Emotional Vulnerability

The logic of personalization further entrenches this profit-through-engagement dynamic. Algorithms that simulate empathy or tailor content to user affective states are framed as therapeutic tools, offering frictionless, always-available companionship [75]. AI-based chatbots and digital humans are explicitly designed to make users feel heard and supported, traits widely identified as effective in reducing the perception of loneliness [75]. While some studies show digital interventions can provide temporary relief, their effects are often “short-lived” [16]. Quantitative evidence from Maples et al. [76] and Magid et al. [63] suggests that while such tools may temporarily improve mood or reduce distress, they often fail to sustain long-term social connection, echoing the paradox described above. They are noted for not providing “real human interaction” and, thus, “cannot replace human contact,” failing to reduce social disconnectedness on a long-term basis [16]. This suggests that current digital treatments act as surface-level rather than long-term solutions. Yet these interactions often constitute the above-mentioned “illusion of recognition”: a simulation of social reciprocity without true mutuality or intersubjectivity [50]. Qualitative studies such as Meadows and Hine [57] and Fullam [55] show how users of mental health chatbots experience these systems not simply as tools but as affective environments that reshape expectations of intimacy and care. Rather than disrupting isolation, they reorganize it and transform loneliness from a felt absence of connection into a perpetual state of digitally mediated pseudo-connection. This is the paradox of affective AI within capitalist infrastructures: it relieves symptoms while sustaining the underlying condition.

### 6.3. Algorithmic Affordances and the Redefinition of Connection

Algorithmic infrastructures displace the role of embodied, face-to-face communication by offering convenient, scalable substitutes [77]. Digital platforms are often positioned as solutions to access barriers by providing support to users in remote regions, with limited mobility, or lacking traditional mental health resources; however, their increasing integration into daily life risks supplanting, rather than supplementing, high-quality human relationships [25,48,77,78]. The very affordances that make digital interventions scalable (i.e., predictability, availability, and affective responsiveness) can also produce dependency, flatten emotional complexity, and disincentivize engagement with the "inherent messiness" of real-world relationality [48,57,61,68]. What emerges is not just the erosion of social skill or opportunity but the redefinition of connection itself according to the logics of responsiveness, efficiency, and user retention.

### 6.4. Extraction, Bias, and the Medicalized Reframing of Loneliness

These background actors are also mechanisms of classification and control. Through digital phenotyping and the collection of granular behavioural data, platforms actively structure users’ engagement with emotional experiences [18]. Data become a currency that is harvested, analyzed, and often commodified to predict user states, automate interventions, and refine engagement strategies [79]. This orientation positions the user simultaneously as a subject in distress and a data source to be mined, rendering the affective experience of loneliness legible primarily as a behavioural variable within a feedback system. While such models purport to offer care, they do so within architectures that are fundamentally extractive, opaque, and profit driven [79].

Trained on narrow datasets and optimized for broad-market appeal, these systems are also embedded with epistemic and representational limitations. AI systems risk reinforcing dominant cultural norms, societal biases, and normative assumptions about intimacy and relational need [61,79]. If left unexamined, these biases can reproduce exclusionary dynamics, marginalizing users whose identities, values, or communication styles fall outside those anticipated by the system. What results is not a universal tool for connection but a highly contingent intervention shaped by the market’s image of loneliness and the individual it imagines as its subject.

Finally, the structural logics of platform capitalism encourage the previously described medicalized and individualized framing of loneliness. Digital loneliness interventions often locate the “problem” within the individual that positions users as deficient or dysregulated subjects in need of affective optimization [62]. This responsibility aligns with broader neoliberal discourses of self-management, in which structural determinants of disconnection (e.g., precarious labour, urban alienation, racialized exclusion, or defunded public infrastructure) are rendered invisible. In treating loneliness as a symptom to be managed through personalized digital solutions, such systems deflect attention from the social, political, and economic conditions that produce it [62]. Public health becomes reframed as a technological marketplace while systemic reform is replaced by therapeutic interface.

## 7. Public Health and Technological Design

### 7.1. The Systemic Framing of Loneliness in Public Health and Design

The convergence of public health and technology by way of design reflects a growing recognition that loneliness is not only a psychological state but also a systemic and technologically mediated phenomenon [9,15,49]. Policy analyses increasingly highlight that digital tools are being positioned as a part of national strategies to address loneliness, embedding technological interventions within broader public health frameworks [2,3,4,7]. While both public health and design fields acknowledge AI’s potential to mediate social connection, empirical studies also show that algorithmic systems can perpetuate or even produce loneliness through their underlying logics, reshaping relational norms and displacing authentic human interaction [17,50].

Public health institutions have increasingly identified loneliness as a pervasive and urgent public health concern, particularity following the COVID-19 pandemic, associated with heightened risks of depression, anxiety, cardiovascular disease, cognitive decline, and premature mortality [1,6,8,10,12,13,14,15,16]. Recent epidemiological studies extend this picture: Fahy and Barry [65] show how online social capital interacts with loneliness, while Infurna et al. [80] find that loneliness levels in midlife have risen over decades, especially in digitally saturated contexts. Leading bodies such as the World Health Organization and the U.S. Surgeon General have positioned loneliness as a global crisis, calling for systemic responses and explicitly recommending the development of “pro-connection technology” and the exploration of digital interventions [61,62,81,82]. These imperatives, situated within a broader framework of the social determinants of health, have catalyzed the design field to develop responsive technologies aimed at connection, care, and accessibility. 

### 7.2. Digital Interventions and Conditional Promises of Connection

Despite this review’s already-significant critiques of technology-based public health interventions, both fields also recognize the potential of AI to mediate connection under specific conditions. Digital technologies can address access barriers to traditional mental health care, offering support to those facing geographic, financial, or mobility constraints [83,84]. Virtual meetups, telehealth, and digital peer support networks have proven particularly valuable during moments of crisis, such as the COVID-19 pandemic [48]. Some AI companions and platforms incorporate therapeutic frameworks like Cognitive Behavioural Therapy (CBT) or narrative coaching, providing structured support that may alleviate subjective feelings of loneliness in the short term [54,76]. Certain interventions have been designed with transitional intent such as helping users develop communicative competencies or encouraging re-engagement with real-world social environments through hybrid tools such as location-based games or social prompts [67].

### 7.3. Designing for Relational Justice: Toward Ethical and Inclusive Systems

What emerges from this intersection is a call for ethically grounded, human-centered design [13,48]. Ethical analyses such as Jecker et al. [61] argue for policy safeguards when deploying digital solutions for social support, noting that the capacity for simulated empathy demands corresponding protections against misuse. Public health perspectives insist that loneliness must be understood as a socially patterned and structurally produced phenomenon, not merely a symptom to be managed through individual digital use [49,52,54,61]. This orientation challenges the design field to develop interventions that resist individualization, foreground user autonomy, and account for social, economic, and cultural inequities. Design responses should be informed by participatory methods, community-based research, and ethical foresight, with particular interest to those that center accessibility, transparency, and inclusivity, which can help mitigate some of the harms introduced by commercial and algorithmic systems [52,54,61,65].

At the policy level, both fields advocate for increased regulation, interdisciplinary oversight, and long-term evaluation of digital mental health interventions [2,4,14,85]. There is growing consensus that ethical and technical governance must be instituted to protect vulnerable populations from manipulation, surveillance, or further marginalization [54,57,79,86]. This is further coupled with an urgent need to move beyond short-term assessments of efficacy to examine the long-term psychosocial effects of digital interactions on loneliness, relational depth, and communal cohesion [57,79,82].

The connection between public health and design is not simply one of task delegation, where health systems define problems and designers generate solutions, but one of epistemological and ethical entanglement. Both fields must engage in ongoing dialogue to interrogate how digital infrastructures are conceptualized, deployed, and experienced. From a design justice perspective, Pendse et al. [79] call for decolonial approaches that challenge dominant narratives and ensure technologies are shaped by, and accountable to, the communities they serve. Addressing loneliness in the context of AI requires not only technological innovation but also a collective commitment to reimagining relationality, accountability, and care in an era shaped by algorithmic systems.

## 8. Digital Public Health Design Framework

The preceding sections traced how the four interconnected domains of hegemonic digital design paradigms, algorithmic mediation, and public health framing shape the experience of loneliness and the interventions proposed to address it. The structural critiques outlined in Section 5 identify the commercial and algorithmic logics that undermine relational depth, pointing to the need for relational personalization and digital well-being by design. Section 6’s analysis of algorithmic mediation highlights the narrowing of informational and emotional experience, underscoring the importance of hybrid and real-world connection and adaptive, non-coercive support. Behavioural science offers several design strategies to support adaptive, non-coercive interventions: Mele et al. [23] describe “smart nudging” as a way to co-create value with users, Joachim et al. [87] apply nudge theory to AI-driven health platforms, and Chiam et al. [88] demonstrate how algorithmic nudging can be personalized to health outcomes while maintaining transparency. Section 7’s integration of public health perspectives calls for participatory, equitable and accountable approaches. Together, these thematic insights directly inform the ethical design priorities that follow.

To address loneliness as both a public health crisis and a technologically mediated condition, this review proposes a human-centered and ethically grounded framework for the design of AI and digital interventions. Rather than reproducing the logic of behavioural enclosure, this framework supports relational autonomy, structural responsiveness, and ethical accountability. Rooted in public health imperatives, human-centered design principles, and critical analyses of platform capitalism, it offers an alternative to engagement-driven models that often exacerbate the very issues they seek to resolve [49]. Instead of managing the symptoms of loneliness, it foregrounds the structural and systemic conditions that produce and perpetuate it.

The framework begins with a philosophical reorientation. Loneliness should not be treated solely as a pathological deficit to be remedied through technological substitution. Instead, it must be understood as a relational signal, an embodied, affective form of attunement that indicates unmet needs for social connection [11,12,13]. In this reframing, digital tools are positioned as facilitators of social repair rather than surrogates for social life. This shift is rooted in a model of relational autonomy, which is the understanding that autonomy is not the absence of dependence but the ability to act meaningfully within a network of social, cultural, and structural relationships [41,42,45]. Unlike individualistic models of choice, relational autonomy recognises that agency is shaped by context, care, and reciprocity [42]. This demands recognition of the structural determinants shaping experiences of loneliness, including economic precarity, housing insecurity, systemic discrimination, and unequal access to care [13,15,61,62]. Technology, in this context, should be designed to support human flourishing in culturally and materially specific ways. It requires an ethical shift away from replacement logics toward the augmentation of human relationships. AI systems must scaffold, prompt, and gently encourage connection, not reroute relational energy into synthetic stand-ins. A decolonial, context-sensitive approach is essential, one that centers lived experience, acknowledges cultural specificity, and actively disrupts power asymmetries in how technologies are imagined, designed, and accessed [79].

At the heart of this framework lies a set of ethical principles, adapted from Löchner et al.’s TEQUILA model (Trust, Evidence, Quality, Usability, Interests, Liability, Accreditation) [18], and revised to address the specific challenges of AI in public health. Trust must be earned through transparent data governance, continuous and informed consent, and clear communication of the capacities and limitations of artificial agents. Users should retain full control over their emotional, behavioural, and biometric data, and transparency must include explicit disclosures about AI-generated content and simulated empathy.

High standards of evidence and quality are also critical. Interventions must be rigorously evaluated through long-term, methodologically robust studies across diverse populations [18,61,82]. Success should be measured not by engagement metrics but by their capacity to foster emotional resilience, deepen social ties, and promote community integration [61]. Clinical accreditation and regulatory oversight are essential for systems with therapeutic claims, alongside meaningful human oversight for any AI making interpretive judgments about users’ mental health [82].

Equally vital is a participatory design process grounded in human-centered values. End users, mental health practitioners, and public health experts must be involved throughout development, from needs assessment to outcome evaluation [14,48,79,87]. Systems should empower users by promoting autonomy and discouraging dependency, avoiding manipulative design patterns that exploit vulnerability [25,48,73]. Algorithmic systems must be audited for bias and actively corrected to avoid reproducing structural inequities related to race, gender, disability, or class [61,79]. Equity must be embedded in the design logic from the outset.

Clear lines of accountability must be maintained throughout the entire lifecycle of digital interventions. Responsibility for outcomes, from data handling to harm mitigation, cannot be diffused through technical abstraction [18,54,89]. The myth of algorithmic neutrality must be replaced with governance structures that acknowledge the political and material stakes of AI systems. Certification protocols are particularly important for platforms and agents performing quasi-clinical functions in mental health.

**Figure 2:**
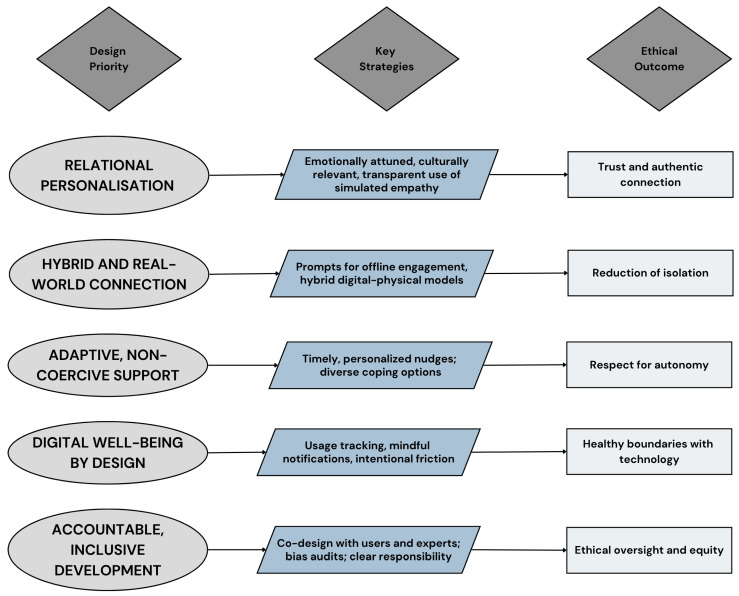
Ethical outcomes of design priorities for digital interventions addressing loneliness.

While all digital interventions must be evaluated for bias and equitable access, AI companions require safeguards around emotional simulation and user dependency. To operationalize these ethical commitments, Figure 2 identifies the following key functional priorities:

Personalization should be used not to increase retention but to ensure relevance, emotional recognition, and cultural sensitivity [14,48,82,87]. While emotionally intelligent responses and simulated empathy can help users feel recognized, these functions must remain transparent to avoid misidentification or confusion. In the case of social robots, nonverbal cues such as gaze or gesture may enhance interaction but must be carefully calibrated to avoid the phenomenon commonly known as the uncanny valley, which can disrupt trust and emotional resonance.

Digital systems should also reinforce real-world connection. Features such as prompts to connect with friends, community event suggestions, and referrals to local resources help bridge online engagement with embodied sociality [14,48,82,90]. Hybrid models that integrate online tools with offline support can mitigate the risk of relational displacement. Adaptive systems, such as Just-in-Time Adaptive Interventions (JITAIs), can offer timely and context-sensitive nudges that promote well-being without coercion [21,23,25]. These should encourage, not compel, social activity, movement, and emotional regulation. A practical illustration of the “hybrid and real-world connection” priority is the +*Connect* smartphone application, a positive psychology-based program co-designed with young people who self-identified as experiencing loneliness [14]. The intervention delivered daily digital content over six weeks, including short videos, reflective exercises, and “real-world” missions, which prompt users to initiate or deepen social interactions offline [14]. In a pilot randomized controlled trial involving young adults both with and without social anxiety disorder, +*Connect* achieved high rates of engagement and yielded measurable improvements in self-reported social connectedness and reductions in loneliness [14]. Qualitative feedback highlighted the value of combining asynchronous, self-paced digital learning with structured encouragement to practice skills in everyday life, supporting the idea that digital public health interventions can be designed to bridge online engagement with offline relationship building in ways that are perceived as supportive rather than intrusive [14].

Rather than promoting a universalized vision of healthy behaviour, interventions must recognize and support the diverse coping strategies users already employ. From creative expression and distraction to introspection and social withdrawal, digital tools must be flexible enough to accommodate varied paths through loneliness [25,48]. Systems should also help prevent overdependence: tools such as usage dashboards, mindful notification settings, and intentional design friction can support digital well-being and more reflective engagement [48,73]. Pretolesi et al. [91] explore user preferences for customization, Janković et al. [92] show how adaptive notifications can improve engagement in behaviour-change apps, and Auf et al. [60] examine gamification techniques that balance motivation with user autonomy. Crucially, interventions must maintain a clear boundary between technological support and relational replacement while maintaining trust through empathy [48,73,93,94].

Meaningful solutions to loneliness require sustained and interdisciplinary collaboration between fields, humans, and machines [48,95,96]. Designers, engineers, social scientists, ethicists, clinicians, and policymakers must work together from ideation to implementation. Feedback loops must be built into these systems, allowing for iterative refinement based on lived experience and emergent harms. Longitudinal studies are needed to assess not only clinical efficacy but also the social and political consequences of intervention. Policy frameworks must promote equitable access, mandate accountability, and protect vulnerable users from exploitation.

Addressing loneliness through AI and digital public health tools requires a multidimensional approach, philosophical, ethical, functional, and political. By resisting reductive engagement metrics and centering relational integrity, this framework offers a path toward technologies that support connection, compassion, and human flourishing in complex and context-specific ways.

## 9. Conclusions

As loneliness emerges as a defining public health challenge of the digital age, it becomes increasingly urgent to examine not only the individual experiences of disconnection but also the technological, economic, and epistemological systems that shape them. This review has argued that loneliness must be understood as both a socially embedded condition and a logical outcome of hegemonic design paradigms, particularly those structured by the logics of platform capitalism and algorithmic control. The very infrastructures that promise to alleviate loneliness, such as AI companions, wellness apps, and digital health tools, are embedded in the logics of platform capitalism and engagement-driven design, monetizing attention [26,32]. The intimacy through simulated empathy replaces genuine social connection with commercially mediated interactions [15,42,63].

By tracing the evolution of gatekeeping from a human editorial process to an opaque, computational logic embedded in algorithmic infrastructures, the analysis has shown how visibility, emotion, and legitimacy are increasingly governed by profitability and predictive accuracy. Within this paradigm, loneliness is not merely mediated; it is produced. In this new algorithmic epistemology, emotional profiling replaces public discourse, and behavioral nudging becomes a quiet, ambient form of governance [20]. This review proposes that loneliness is not only framed and mediated but produced through what might be called “algorithmic epistemology,” a logic of knowing grounded in engagement-generated, affect-oriented, and predictive algorithms [89,95]. As Maalsen [89] shows, algorithmic systems reconfigure epistemic landscapes by actively shaping how we come to know and interpret social and spatial realities. This epistemological shift is further underscored by Loosen and Scholl [95], who argue that algorithms function as observing systems that construct meaning rather than passively reflect it, and by Milano et al. [96], who demonstrate how algorithmic profiling can fragment individuals’ interpretive capacities, limiting their ability to share experiences and resist system-driven sense making. These systems do not just reflect user reality; they recalibrate it. What is most seen becomes what is most real.

This review recognizes that digital interventions vary widely in function and design; however, the shared infrastructure of algorithmic governance and behavioural design necessitates a critical lens across domains. Digital interventions are not inherently harmful, but when embedded in systems optimized for surveillance, scalability, and retention, they risk becoming part of the problem they seek to solve. If designed without critical reflection, they may individualize systemic issues, reinforce existing inequities, and offer only superficial comfort in place of sustained, structural solutions. Addressing loneliness, therefore, demands more than innovation; it requires transformation of design priorities, business models, regulatory structures, and public imaginaries.

The framework proposed in this review offers a path forward, one grounded in public health ethics, human-centered design, and political accountability. By reframing loneliness as a relational signal rather than a personal failure, and by designing AI tools to augment rather than replace human connection, it is possible to develop digital interventions that respect autonomy, enable genuine engagement, and resist exploitative logics. Such tools must be transparent, context-sensitive, and continually evaluated for their long-term impacts, not just on individuals but on the social fabrics they inhabit.

Designing for loneliness is not only a technological challenge; it is a moral and cultural one. It calls for interdisciplinary collaboration, policy reform, and, above all, a renewed commitment to human dignity in an age of machine mediation. In reimagining how we relate to both each other and the systems we build, we are not simply addressing loneliness; we are redefining the terms of connection itself.

## 10. Limitations and Future Directions

A meta-narrative review, by design, does not aim for exhaustive inclusion of all possible studies, nor does it provide a quantitative meta-analysis. Instead, it prioritizes conceptual depth and cross-disciplinary dialogue. As such, some relevant empirical studies may not have been captured, particularly outside the time frame or language searched. The focus on English-language sources and the reliance on available database indexing may also introduce selection bias. All conclusions and the framework presented here should be read as integrative, rather than definitive, offering a foundation for further empirical testing and refinement.

The growing integration of AI-driven systems into loneliness interventions presents a critical area for future research at the intersection of technology, public health, and relational design. One area of note is social robotics and digital humans. As these fields evolve beyond assistive functions toward roles as emotionally responsive companions, they raise important questions about the reconfiguration of care, connection, and intimacy in technologically mediated contexts. Future research should expand Freitas et al.’s [17] work, which provides evidence that AI companions can reduce loneliness in controlled settings, as well as the work of Mahajan [54], who explores their potential for integration into family-like roles, and Lynch et al. [90], who cautions that such automation can displace human affective labour.

Future studies should investigate how affective AI systems simulate empathy, personalize interaction, and offer scalable support for structurally underserved populations. Equally important is the need to assess the long-term psychosocial impacts of such systems, including risks of dependency, emotional flattening, and the commodification of human relationships. Work by Sharma et al. [93] and Pralat et al. [94] demonstrates that human–AI collaboration can foster more empathetic, trust-building interactions, findings which could inform ethical frameworks and technical implantation.

This research agenda must also address ethical and governance concerns, including consent, bias mitigation, and accountability, in the collection and use of affective data. Frameworks such as TEQUILA [12] offer starting points for evaluating responsible deployment but require empirical testing and contextual adaptation.

As AI companions increasingly blur the boundaries between care, commerce, and relationality, interdisciplinary research is needed to ensure these systems enhance rather than erode the conditions for meaningful social connection. The challenge lies not only in developing technically sophisticated tools but in reimagining digital interventions that prioritize human dignity, autonomy, and structural equity.

## Figures and Tables

**Table 1:** Ethical design priorities for digital interventions addressing loneliness.

Research Tradition	Framing of Loneliness + Technology	Methodological Orientation	Design Implications
Public Health	Technology positioned as a scalable mechanism to address loneliness, primarily treated as a modifiable health risk.	Epidemiological surveys, longitudinal studies, validated psychometric scales, intervention trials.	Integrate structural critiques into intervention design to avoid treating loneliness solely as an individual pathology.
Behavioural Science & Psychology	Technology as a medium for behaviour change, social skills training, and cognitive reframing to reduce loneliness.	Behaviour change theory, CBT, nudge theory, experimental and quasi-experimental studies.	Ensure long-term relational outcomes by combining behavioural strategies with safeguards against dependency and over-reliance
HCI/Design	Technology as a sociotechnical system whose affordances shape relational depth, agency, and connection quality.	User-centered and participatory design, affordance theory, systems thinking, usability studies.	Prioritizes hybrid online-offline connections, design “positive friction” and preserve user agency in relational contexts.
Communication & Media	Technology as embedded in political-economic systems that commodify connection and influence emotional life.	Political economy of media, gatekeeping theory, critical discourse analysis.	Address platform logics and governance structures to design interventions that resist commodification and structural disconnection.

## Data Availability

No new data were created or analyzed in this study. Data sharing is not applicable to this article.

## Supplementary Materials

The following supporting information can be downloaded at: https://www.mdpi.com/article/10.3390/ijerph22091394/s1, Table S1: Classification of articles for analysis.
