# Supplementary material for: Loneliness by Design: The Structural Logic of Isolation in Engagement-Driven Systems
Source: Int J Environ Res Public Health. 2025 Sep 6;22(9):1394. doi: 10.3390/ijerph22091394 (PMC12470018; doi:10.3390/ijerph22091394)
Supplement: Supplementary file 1 [file ijerph-22-01394-s001.zip › ijerph-3751322-supplementary.pdf]

Table S1: Classification of articles for analysis

Thematics Domains and Sub-Domains Legend:

1. The Hegemony of Digital Design Paradigms
    a. Hegemonic Digital Infrastructures and the Structuring of Loneliness
    b. Simulated Intimacy and the Erosion of Relational Depth
    c. Design Affordances, Sensory Deficits, and Affective Disconnection
    d. Individualisation, Medicalization, and the Obfuscation of Structural Causes
2. Algorithmic Infrastructures as Mediator and Producer
    a. Technology as Mediator
    b. Technology as Producer
3. Structural Logics of Platform Capitalism and Algorithmic Control
    a. Platform Capitalism and the Infrastructure of Loneliness
    b. Affective AI and the Commodification of Emotional Vulnerability
    c. Algorithmic Affordances and the Redefinition of Connection
    d. Extraction, Bias, and the Medicalized Reframing of Loneliness
4. Public Health and Technological Design
    a. The Systemic Framing of Loneliness in Public Health and Design
    b. Digital Interventions and Conditional Promises of Connection
    c. Designing for Relational Justice: Toward Ethical and Inclusive Systems
5. Digital Public Health Design Framework

Number	Title	Tradition	Thematic Domain(s)	Sub-Domain(s)
			Public Health and Technological Design	The Systemic Framing of Loneliness in Public Health and Design
1	Pandemics as Socio-Political Phenomena	Public Health and Clinical Epidemiology		
2	Addressing loneliness and social isolation in 52 countries: a		The Hegemony of Digital Design Paradigms	Hegemonic Digital Infrastructures and the Structuring of Loneliness

	scoping review of National policies	Public Health and Clinical Epidemiology	Public Health and Technological Design	The Systemic Framing of Loneliness in Public Health and Design<br><br>Designing for Relational Justice: Toward Ethical and Inclusive Systems
3	Technology and older adults in British loneliness policy and political discourse	Public Health and Clinical Epidemiology	Public Health and Technological Design	The Systemic Framing of Loneliness in Public Health and Design
4	Problematizing loneliness as a public health issue: an analysis of policy in the United Kingdom	Public Health and Clinical Epidemiology	Public Health and Technological Design	Designing for Relational Justice: Toward Ethical and Inclusive Systems
6	How the Pandemic Has Deepened an Epidemic of Loneliness and What We Can Do About It	Behavioural Science and Psychology,	Public Health and Technological Design	The Systemic Framing of Loneliness in Public Health and Design
7			The Hegemony of Digital Design Paradigms	Hegemonic Digital Infrastructures and the Structuring of Loneliness

	Public policy and the reduction and prevention of loneliness and social isolation	Public Health and Clinical Epidemiology	Algorithmic Infrastructures as Mediator and Producer	Technology as Mediator
			Public Health and Technological Design	The Systemic Framing of Loneliness in Public Health and Design
8	Diagnostic and statistical manual of mental disorders: DSM-5-TR	Public Health and Clinical Epidemiology		
			Public Health and Technological Design	The Systemic Framing of Loneliness in Public Health and Design
9	Loneliness Is Associated with Depressive Affect, But Not with Most Other Symptoms of Depression in Community-Dwelling Individuals: A Network Analysis. International Journal of Environmental Research and Public Health	Public Health and Clinical Epidemiology		
			Public Health and Technological Design	The Systemic Framing of Loneliness in Public Health and Design
10	Loneliness: Clinical Import and Interventions	Behavioural Science and Psychology,		
			Public Health and Technological Design	The Systemic Framing of Loneliness in Public Health and Design
11	Understanding the development of chronic loneliness in youth	Behavioural Science and Psychology,		

			Digital Public Health Design Framework	
12	Loneliness or Solitude: Which Will We Experience?	Behavioural Science and Psychology,		
			Public Health and Technological Design	The Systemic Framing of Loneliness in Public Health and Design
			Digital Public Health Design Framework	
13	Canadian perspectives on loneliness; digital communication as meaningful connection	Communication and Media Studies.	The Hegemony of Digital Design Paradigms	Hegemonic Digital Infrastructures and the Structuring of Loneliness<br><br>Design Affordances, Sensory Deficits, and Affective Disconnection
			Algorithmic Infrastructures as Mediator and Producer	
			Public Health and Technological Design	The Systemic Framing of Loneliness in Public Health and Design<br><br>Designing for Relational Justice: Toward Ethical and Inclusive Systems
			Digital Public Health Design Framework	
14	A Pilot Digital Intervention Targeting Loneliness in Youth Mental Health	Public Health and Clinical Epidemiology	The Hegemony of Digital Design Paradigms	Hegemonic Digital Infrastructures and the Structuring of Loneliness
			Public Health and Technological Design	The Systemic Framing of Loneliness in Public Health and Design

				Designing for Relational Justice: Toward Ethical and Inclusive Systems
15	Digital Companions for Well-being: Challenges and Opportunities	Human-Computer Interaction & Design	The Hegemony of Digital Design Paradigms	Hegemonic Digital Infrastructures and the Structuring of Loneliness<br><br>Simulated Intimacy and the Erosion of Relational Depth
			Public Health and Technological Design	The Systemic Framing of Loneliness in Public Health and Design
			Digital Public Health Design Framework	
16	Evaluation of the Effectiveness of Digital Technology Interventions to Reduce Loneliness in Older Adults: Systematic Review and Meta-analysis	Public Health and Clinical Epidemiology	The Hegemony of Digital Design Paradigms	Hegemonic Digital Infrastructures and the Structuring of Loneliness<br><br>Individualisation, Medicalization, and the Obfuscation of Structural Causes
			Structural Logics of Platform Capitalism and Algorithmic Control	Affective AI and the Commodification of Emotional Vulnerability
			Public Health and Technological Design	The Systemic Framing of Loneliness in Public Health and Design
17	AI Companions Reduce Loneliness	Human-Computer Interaction & Design	The Hegemony of Digital Design Paradigms	Hegemonic Digital Infrastructures and the Structuring of Loneliness<br><br>Simulated Intimacy and the Erosion of Relational Depth

			Algorithmic Infrastructures as Mediator and Producer	Technology as Mediator
			Public Health and Technological Design	The Systemic Framing of Loneliness in Public Health and Design
18	Digital interventions in mental health: An overview and future perspectives	Public Health and Clinical Epidemiology	The Hegemony of Digital Design Paradigms	Hegemonic Digital Infrastructures and the Structuring of Loneliness
			Structural Logics of Platform Capitalism and Algorithmic Control	Platform Capitalism and the Infrastructure of Loneliness<br><br>Extraction, Bias, and the Medicalized Reframing of Loneliness
19	Apps for Mental Health: An Evaluation of Behavior Change Strategies and Recommendations for Future Development	Human-Computer Interaction & Design	The Hegemony of Digital Design Paradigms	Hegemonic Digital Infrastructures and the Structuring of Loneliness
20	Nudging Algorithms by Influencing Human Behavior: Effects of Encouraging Fact-Checking on News Rankings	Human-Computer Interaction & Design		
			Structural Logics of Platform Capitalism and Algorithmic Control	Platform Capitalism and the Infrastructure of Loneliness

21	‘I guess I was surprised by an app telling an adult they had to go to bed before half ten’: a phenomenological exploration of behavioural ‘nudges’	Behavioural Science and Psychology,	Digital Public Health Design Framework	
22	The growing field of digital psychiatry: current evidence and the future of apps, social media, chatbots, and virtual reality	Public Health and Clinical Epidemiology	Digital Public Health Design Framework	
23	Smart nudging: How cognitive technologies enable choice architectures for value co-creation	Behavioural Science and Psychology,	Algorithmic Infrastructures as Mediator and Producer	Technology as Mediator
			Digital Public Health Design Framework	
24	The Feel of Algorithms	Communication and Media Studies.	Algorithmic Infrastructures as Mediator and Producer	
			Structural Logics of Platform Capitalism and Algorithmic Control	Platform Capitalism and the Infrastructure of Loneliness
25	Designing meaningful future digital interactions: fostering	Human-Computer Interaction & Design	The Hegemony of Digital Design Paradigms	Design Affordances, Sensory Deficits, and Affective Disconnection

	wellbeing to reduce techno-induced stress			Individualisation, Medicalization, and the Obfuscation of Structural Causes
			Structural Logics of Platform Capitalism and Algorithmic Control	Algorithmic Affordances and the Redefinition of Connection
26	In AI We Trust: Power, Illusion and Control of Predictive Algorithms	Communication and Media Studies.	Structural Logics of Platform Capitalism and Algorithmic Control	Platform Capitalism and the Infrastructure of Loneliness
27	Gatekeeping Theory	Communication and Media Studies.	Algorithmic Infrastructures as Mediator and Producer	Technology as Producer
28	The Politics of ‘Platforms	Communication and Media Studies.	Algorithmic Infrastructures as Mediator and Producer	Technology as Producer
29				

	Do Not Recommend? Reduction as a Form of Content Moderation	Communication and Media Studies.	Algorithmic Infrastructures as Mediator and Producer	Technology as Producer
30	Data Colonialism: Rethinking Big Data's Relation to the Contemporary Subject	Communication and Media Studies.	The Hegemony of Digital Design Paradigms	Hegemonic Digital Infrastructures and the Structuring of Loneliness
31	The Age of Surveillance Capitalism: The Fight for a Human Future at the New Frontier of Power	Communication and Media Studies.	The Hegemony of Digital Design Paradigms	Hegemonic Digital Infrastructures and the Structuring of Loneliness<br><br>Design Affordances, Sensory Deficits, and Affective Disconnection
			Algorithmic Infrastructures as Mediator and Producer	Technology as Producer
			Structural Logics of Platform Capitalism and Algorithmic Control	Platform Capitalism and the Infrastructure of Loneliness
32	Platform Capitalism	Communication and Media Studies.	The Hegemony of Digital Design Paradigms	Design Affordances, Sensory Deficits, and Affective Disconnection<br><br>Individualisation, Medicalization, and the Obfuscation of Structural Causes

			Algorithmic Infrastructures as Mediator and Producer	Technology as Producer
			Structural Logics of Platform Capitalism and Algorithmic Control	Platform Capitalism and the Infrastructure of Loneliness
			[redacted]	[redacted]
			[redacted]	[redacted]
33	“Not This One”: Social Movements, the Attention Economy, and Microcelebrity Networked Activism	Communication and Media Studies.	[redacted]	[redacted]
			Algorithmic Infrastructures as Mediator and Producer	Technology as Producer
			[redacted]	[redacted]
			[redacted]	[redacted]
			[redacted]	[redacted]
34	The challenges of platform capitalism: Understanding the logic of a new business model	Communication and Media Studies.	The Hegemony of Digital Design Paradigms	Design Affordances, Sensory Deficits, and Affective Disconnection
			[redacted]	[redacted]
			[redacted]	[redacted]
			[redacted]	[redacted]
			[redacted]	[redacted]
35	Alone Together: Why We Expect More from Technology and Less from Each Other	Communication and Media Studies.	The Hegemony of Digital Design Paradigms	Hegemonic Digital Infrastructures and the Structuring of Loneliness
			[redacted]	[redacted]
			[redacted]	[redacted]
			[redacted]	[redacted]
			[redacted]	[redacted]
36	Cold Intimacies: The Making of Emotional Capitalism	Communication and Media Studies.	[redacted]	[redacted]
			[redacted]	[redacted]

			Structural Logics of Platform Capitalism and Algorithmic Control	Platform Capitalism and the Infrastructure of Loneliness
			Algorithmic Infrastructures as Mediator and Producer	Technology as Producer
37	The Filter Bubble: What The Internet Is Hiding From You	Communication and Media Studies.		
			The Hegemony of Digital Design Paradigms	Hegemonic Digital Infrastructures and the Structuring of Loneliness
41	Artificial intelligence as digital agency	Human-Computer Interaction & Design	Digital Public Health Design Framework	
			The Hegemony of Digital Design Paradigms	Hegemonic Digital Infrastructures and the Structuring of Loneliness
			Algorithmic Infrastructures as Mediator and Producer	Technology as Producer
42	Live Like Nobody Is Watching: Relational Autonomy in the Age of Artificial Intelligence Health Monitoring	Human-Computer Interaction & Design		
			The Hegemony of Digital Design Paradigms	Hegemonic Digital Infrastructures and the Structuring of Loneliness
43	From Entity to Relation? Agency in the Era of Artificial Intelligence	Communication and Media Studies.		

44	A Conversational Artificial Intelligence Agent for a Mental Health Care App: Evaluation Study of Its Participatory Design	Human-Computer Interaction & Design	The Hegemony of Digital Design Paradigms	Hegemonic Digital Infrastructures and the Structuring of Loneliness
45	Relational agency: Relational sociology, agency and interaction	Communication and Media Studies.	The Hegemony of Digital Design Paradigms<br>Digital Public Health Design Framework	Hegemonic Digital Infrastructures and the Structuring of Loneliness
46	What kind of a problem is loneliness? Representations of connectedness and participation from a study of telepresence technologies in the UK	Public Health and Clinical Epidemiology	The Hegemony of Digital Design Paradigms	Hegemonic Digital Infrastructures and the Structuring of Loneliness<br>Design Affordances, Sensory Deficits, and Affective Disconnection
47		Human-Computer Interaction & Design	The Hegemony of Digital Design Paradigms	Hegemonic Digital Infrastructures and the Structuring of Loneliness

	Exploring a Behavioral Model of “Positive Friction” in Human-AI Interaction			Design Affordances, Sensory Deficits, and Affective Disconnection
48	Isolated Circuits: Human Experience and Robot Design for the Future of Loneliness	Communication and Media Studies.	The Hegemony of Digital Design Paradigms	Hegemonic Digital Infrastructures and the Structuring of Loneliness<br><br>Simulated Intimacy and the Erosion of Relational Depth
			Algorithmic Infrastructures as Mediator and Producer	
			Structural Logics of Platform Capitalism and Algorithmic Control	Algorithmic Affordances and the Redefinition of Connection
			Public Health and Technological Design	Digital Interventions and Conditional Promises of Connection<br><br>Designing for Relational Justice: Toward Ethical and Inclusive Systems
			Digital Public Health Design Framework	
49	The Ambivalent Impact of Digital Technology on Loneliness: Navigating Connection and Isolation in the Digital Age	Behavioural Science and Psychology,	The Hegemony of Digital Design Paradigms	Hegemonic Digital Infrastructures and the Structuring of Loneliness
			Algorithmic Infrastructures as Mediator and Producer	Technology as Producer
			Public Health and Technological Design	The Systemic Framing of Loneliness in Public Health and Design

				Designing for Relational Justice: Toward Ethical and Inclusive Systems
			Digital Public Health Design Framework	
50	Digital loneliness—changes of social recognition through AI companions	Public Health and Clinical Epidemiology	The Hegemony of Digital Design Paradigms	Hegemonic Digital Infrastructures and the Structuring of Loneliness<br><br>Simulated Intimacy and the Erosion of Relational Depth
			Algorithmic Infrastructures as Mediator and Producer	Technology as Mediator<br><br>Technology as Producer
			Structural Logics of Platform Capitalism and Algorithmic Control	Affective AI and the Commodification of Emotional Vulnerability
			Public Health and Technological Design	The Systemic Framing of Loneliness in Public Health and Design<br><br>Digital Interventions and Conditional Promises of Connection
51	The Impact of Social Media Use on Loneliness through an Interpersonal-Connection-Behavior Framework	Communication and Media Studies.	The Hegemony of Digital Design Paradigms	Hegemonic Digital Infrastructures and the Structuring of Loneliness<br><br>Design Affordances, Sensory Deficits, and Affective Disconnection
52	The Allure of Artificial Intimacy: Examining the	Human-Computer Interaction & Design	The Hegemony of Digital Design Paradigms	Simulated Intimacy and the Erosion of Relational Depth

	Appeal and Ethics of Using Generative AI for Simulated Relationships		Algorithmic Infrastructures as Mediator and Producer	Technology as Mediator<br><br>Technology as Producer
			Structural Logics of Platform Capitalism and Algorithmic Control	Platform Capitalism and the Infrastructure of Loneliness
			Public Health and Technological Design	Designing for Relational Justice: Toward Ethical and Inclusive Systems
			[redacted]	[redacted]
53	Frictionlessness: The Silicon Valley Philosophy of Seamless Technology and the Aesthetic Value of Imperfection	Communication and Media Studies.	The Hegemony of Digital Design Paradigms	Simulated Intimacy and the Erosion of Relational Depth
			Algorithmic Infrastructures as Mediator and Producer	Technology as Producer
			[redacted]	[redacted]
54	Beyond Biology: AI as Family and the Future of Human Bonds and Relationships	Behavioural Science and Psychology,	The Hegemony of Digital Design Paradigms	Simulated Intimacy and the Erosion of Relational Depth
			[redacted]	[redacted]
			Public Health and Technological Design	Digital Interventions and Conditional Promises of Connection<br><br>Designing for Relational Justice: Toward Ethical and Inclusive Systems
			[redacted]	[redacted]
55	The social life of mental health chatbots	Human-Computer Interaction & Design	The Hegemony of Digital Design Paradigms	Simulated Intimacy and the Erosion of Relational Depth
			Algorithmic Infrastructures as Mediator and Producer	Technology as Mediator

			Structural Logics of Platform Capitalism and Algorithmic Control	Affective AI and the Commodification of Emotional Vulnerability
			[redacted]	[redacted]
			[redacted]	[redacted]
56	The Engagement-Prolonging Designs Teens Encounter on Very Large Online Platforms	Human-Computer Interaction & Design	The Hegemony of Digital Design Paradigms	Simulated Intimacy and the Erosion of Relational Depth
			Algorithmic Infrastructures as Mediator and Producer	Technology as Producer
			[redacted]	[redacted]
			[redacted]	[redacted]
			[redacted]	[redacted]
57	Entanglements of Technologies, Agency and Selfhood: Exploring the Complexity in Attitudes Toward Mental Health Chatbots	Human-Computer Interaction & Design	The Hegemony of Digital Design Paradigms	Simulated Intimacy and the Erosion of Relational Depth
			[redacted]	[redacted]
			Structural Logics of Platform Capitalism and Algorithmic Control	Affective AI and the Commodification of Emotional Vulnerability<br>Algorithmic Affordances and the Redefinition of Connection
			Public Health and Technological Design	Designing for Relational Justice: Toward Ethical and Inclusive Systems
			[redacted]	[redacted]
58	Engagement, user satisfaction, and the amplification of divisive content on social media	Communication and Media Studies.	The Hegemony of Digital Design Paradigms	Design Affordances, Sensory Deficits, and Affective Disconnection
			Algorithmic Infrastructures as Mediator and Producer	Technology as Producer
			[redacted]	[redacted]
			[redacted]	[redacted]
			[redacted]	[redacted]

59	Characterizing and modeling harms from interactions with design patterns in AI interfaces	Human-Computer Interaction & Design	[redacted]	[redacted]
60	Gamification and Nudging Techniques for Improving User Engagement in Mental Health and Well-being Apps	Human-Computer Interaction & Design	The Hegemony of Digital Design Paradigms	Design Affordances, Sensory Deficits, and Affective Disconnection
			[redacted]	[redacted]
61	Digital Humans to Combat Loneliness and Social Isolation: Ethics Concerns and Policy Recommendations	Communication and Media Studies.	The Hegemony of Digital Design Paradigms	Design Affordances, Sensory Deficits, and Affective Disconnection<br><br>Individualisation, Medicalization, and the Obfuscation of Structural Causes
			Algorithmic Infrastructures as Mediator and Producer	Technology as Producer
			Structural Logics of Platform Capitalism and Algorithmic Control	Platform Capitalism and the Infrastructure of Loneliness<br><br>Algorithmic Affordances and the Redefinition of Connection<br><br>Extraction, Bias, and the Medicalized Reframing of Loneliness
			Public Health and Technological Design	The Systemic Framing of Loneliness in Public Health and Design<br><br>Designing for Relational Justice: Toward Ethical and Inclusive Systems

			Digital Public Health Design Framework	
62	Organised loneliness and its discontents	Behavioural Science and Psychology,	The Hegemony of Digital Design Paradigms	Individualisation, Medicalization, and the Obfuscation of Structural Causes
			Structural Logics of Platform Capitalism and Algorithmic Control	Platform Capitalism and the Infrastructure of Loneliness<br><br>Extraction, Bias, and the Medicalized Reframing of Loneliness
			Public Health and Technological Design	The Systemic Framing of Loneliness in Public Health and Design
			Digital Public Health Design Framework	
63	The Impact of Digital Mental Health Services on Loneliness and Mental Health: Results from a Prospective, Observational Study	Behavioural Science and Psychology,		
			Algorithmic Infrastructures as Mediator and Producer	Technology as Mediator
			Structural Logics of Platform Capitalism and Algorithmic Control	Affective AI and the Commodification of Emotional Vulnerability
64	Social Media Use and Perceived Social Isolation Among Young Adults in the U.S	Behavioural Science and Psychology,		
			Algorithmic Infrastructures as Mediator and Producer	Technology as Mediator<br><br>Technology as Producer
65	Investigating the interplay of loneliness, computer-mediated communication, online social	Public Health and Clinical Epidemiology		
			Algorithmic Infrastructures as Mediator and Producer	Technology as Mediator

	capital, and well-being: insights from a COVID-19 lockdown study			
			Public Health and Technological Design	The Systemic Framing of Loneliness in Public Health and Design<br><br>Designing for Relational Justice: Toward Ethical and Inclusive Systems
66	Computational Propaganda: Political Parties, Politicians, and Political Manipulation on Social Media	Communication and Media Studies.		
			Algorithmic Infrastructures as Mediator and Producer	Technology as Producer
67	"If You Build It, They Will Come." Infrastructure, Hegemonic Transition, and Peaceful Change	Communication and Media Studies.		
			Algorithmic Infrastructures as Mediator and Producer	Technology as Producer
			Public Health and Technological Design	Digital Interventions and Conditional Promises of Connection
68	Between the Self and Signal the Dead Internet & A Crisis of Perception,	Human-Computer Interaction & Design		
			Algorithmic Infrastructures as Mediator and Producer	Technology as Producer
			Structural Logics of Platform Capitalism and Algorithmic Control	Algorithmic Affordances and the Redefinition of Connection
69				

	Algorithmic Harms Beyond Facebook and Google: Emergent Challenges of Computational Agency	Communication and Media Studies.	Algorithmic Infrastructures as Mediator and Producer	Technology as Producer
70	Enclosure 4.0: Seizing Data, Selling Predictions, Scaling Platforms	Communication and Media Studies.		
			Algorithmic Infrastructures as Mediator and Producer	Technology as Producer
71	When artificial intelligence substitutes humans in higher education: the cost of loneliness, student success, and retention	Communication and Media Studies.		
			Algorithmic Infrastructures as Mediator and Producer	Technology as Producer
			Structural Logics of Platform Capitalism and Algorithmic Control	Platform Capitalism and the Infrastructure of Loneliness
72	Breaking the filter bubble: democracy and design	Communication and Media Studies.		
			Algorithmic Infrastructures as Mediator and Producer	Technology as Producer
73	Designing for Digital Well-Being: Applying Behavioral Science to Reduce Tech Addiction	Behavioural Science and Psychology,		

			Structural Logics of Platform Capitalism and Algorithmic Control	Platform Capitalism and the Infrastructure of Loneliness
			[redacted]	[redacted]
			Digital Public Health Design Framework	[redacted]
75	Systematic review and meta-analysis of AI-based conversational agents for promoting mental health and well-being	Public Health and Clinical Epidemiology	[redacted]	[redacted]
			[redacted]	[redacted]
			Structural Logics of Platform Capitalism and Algorithmic Control	Affective AI and the Commodification of Emotional Vulnerability
			[redacted]	[redacted]
			[redacted]	[redacted]
76	Loneliness and suicide mitigation for students using GPT3-enabled chatbots	Behavioural Science and Psychology,	[redacted]	[redacted]
			[redacted]	[redacted]
			Structural Logics of Platform Capitalism and Algorithmic Control	Affective AI and the Commodification of Emotional Vulnerability
			Public Health and Technological Design	Digital Interventions and Conditional Promises of Connection
			[redacted]	[redacted]
77	The Concept of Agency in the Era of Artificial Intelligence: Dimensions and Degrees	Behavioural Science and Psychology,	[redacted]	[redacted]
			[redacted]	[redacted]
			Structural Logics of Platform Capitalism and Algorithmic Control	Algorithmic Affordances and the Redefinition of Connection
			[redacted]	[redacted]
			[redacted]	[redacted]
78	Time Spent Using Digital Technology, Loneliness, and Well-Being Among Three Cohorts of Adolescent Girls and Boys – A Moderated Mediation Analysis	Behavioural Science and Psychology,	[redacted]	[redacted]
			Structural Logics of Platform Capitalism and Algorithmic Control	Algorithmic Affordances and the Redefinition of Connection
			[redacted]	[redacted]

			[redacted]	[redacted]
			[redacted]	[redacted]
			[redacted]	[redacted]
			[redacted]	[redacted]
			Structural Logics of Platform Capitalism and Algorithmic Control	Extraction, Bias, and the Medicalized Reframing of Loneliness
			Public Health and Technological Design	Designing for Relational Justice: Toward Ethical and Inclusive Systems
79	From Treatment to Healing: Envisioning a Decolonial Digital Mental Health	Human-Computer Interaction & Design	[redacted]	[redacted]
			[redacted]	[redacted]
			[redacted]	[redacted]
			[redacted]	[redacted]
			Public Health and Technological Design	The Systemic Framing of Loneliness in Public Health and Design
80	Loneliness in midlife: Historical increases and elevated levels in the United States compared with Europe	Behavioural Science and Psychology,	[redacted]	[redacted]
			[redacted]	[redacted]
			[redacted]	[redacted]
			[redacted]	[redacted]
			Public Health and Technological Design	The Systemic Framing of Loneliness in Public Health and Design<br><br>Designing for Relational Justice: Toward Ethical and Inclusive Systems
82	AI Applications to Reduce Loneliness Among Older Adults: A Systematic Review of Effectiveness and Technologies	Public Health and Clinical Epidemiology	Digital Public Health Design Framework	[redacted]
			[redacted]	[redacted]
			[redacted]	[redacted]
83	Advantages and limitations of Internet-based interventions for common mental disorders	Public Health and Clinical Epidemiology	[redacted]	[redacted]

			Public Health and Technological Design	Digital Interventions and Conditional Promises of Connection
			[redacted]	[redacted]
84	Digital technology for treating and preventing mental disorders in low-income and middle-income countries: a narrative review of the literature	Public Health and Clinical Epidemiology	[redacted]	[redacted]
			[redacted]	[redacted]
			[redacted]	[redacted]
			Public Health and Technological Design	Digital Interventions and Conditional Promises of Connection
			[redacted]	[redacted]
85	Do 21st-century skills make you less lonely? The relation between 21st-century skills, social media usage, and students’ loneliness during the COVID-19 pandemic	Communication and Media Studies.	[redacted]	[redacted]
			[redacted]	[redacted]
			[redacted]	[redacted]
			Public Health and Technological Design	Designing for Relational Justice: Toward Ethical and Inclusive Systems
			[redacted]	[redacted]
86	Designing Human-centered AI for Mental Health: Developing Clinically Relevant Applications for Online CBT Treatment	Human-Computer Interaction & Design	[redacted]	[redacted]
			[redacted]	[redacted]
			[redacted]	[redacted]
			Public Health and Technological Design	Designing for Relational Justice: Toward Ethical and Inclusive Systems
			[redacted]	[redacted]
87	A Nudge-Inspired AI-Driven Health Platform for Self-Management of Diabetes	Human-Computer Interaction & Design	[redacted]	[redacted]
			[redacted]	[redacted]
			[redacted]	[redacted]
			[redacted]	[redacted]
			Digital Public Health Design Framework	[redacted]

88	NudgeRank: Digital Algorithmic Nudging for Personalized Health	Human-Computer Interaction & Design	Digital Public Health Design Framework	
89	Exploring user preferences: customisation and attitudes towards notifications in mobile health and well-being applications	Human-Computer Interaction & Design	Digital Public Health Design Framework	
90	Can Personalization Persuade? Study of Notification Adaptation in Mobile Behavior Change Intervention Application	Behavioural Science and Psychology,	Digital Public Health Design Framework	
91	Algorithmic epistemologies and methodologies: Algorithmic harm, algorithmic care and situated algorithmic knowledges	Communication and Media Studies.	Digital Public Health Design Framework	
92	The Epistemological Dimension of Algorithms	Communication and Media Studies.		

			Digital Public Health Design Framework	
93	Algorithmic profiling as a source of hermeneutical injustice	Communication and Media Studies.	Digital Public Health Design Framework	
94	Robotics, Affective Displacement, and the Automation of Care	Communication and Media Studies.	Digital Public Health Design Framework	
95	Human–AI collaboration enables more empathic conversations in text-based peer-to-peer mental health support	Human-Computer Interaction & Design	Digital Public Health Design Framework	
96	Feeling Understood by AI: How Empathy Shapes Trust and Influences Patronage Intentions in Conversational AI	Human-Computer Interaction & Design	Digital Public Health Design Framework	
